# Plasma Aromatase Activity Index, Gonadotropins and Estrone Are Associated with Frailty Syndrome in Post-Menopausal Women with Breast Cancer

**DOI:** 10.3390/curroncol29030144

**Published:** 2022-03-07

**Authors:** Javier García-Sánchez, Mayra Alejandra Mafla-España, Carlos Tejedor-Cabrera, Olga Avellán-Castillo, María Dolores Torregrosa, Omar Cauli

**Affiliations:** 1 Medical Oncology Department, Doctor Peset University Hospital, 46017 Valencia, Spain; javier.garciasanchez@chwapi.be (J.G.-S.); torregrosa_dol@gva.es (M.D.T.); 2 Medical Oncology Department, Hospital Center of Wallonie Picardy, 7500 Tournai, Belgium; 3 Frailty Research Organized Group, University of Valencia, 46010 Valencia, Spain; maymaes@alumni.uv.es; 4 Department of Nursing, University of Valencia, 46010 Valencia, Spain; carteca@alumni.uv.es (C.T.-C.); olacas@alumni.uv.es (O.A.-C.)

**Keywords:** ageing, breast cancer, biomarker, geriatric evaluation, oncogeriatrics, side effect, androgen, estrogens

## Abstract

Frailty syndrome is associated with poor outcomes, morbidity and premature mortality. We performed a cross-sectional study to evaluate the presence of frailty syndrome based on Fried’s frailty phenotype in post-menopausal women with breast cancer. We further analyzed the association between frailty syndrome with geriatric assessments and the association with the concentration of gonadotropins LH and FSH, estrogens, androgens and the aromatase activity index in the blood. We enrolled 47 post-menopausal women with localized breast cancer (mean age 66.8 ± 1.3 years (range 52–83)) prior to the starting of adjuvant endocrine therapy. Patients were identified as “non-frail” (robust) or “prefrail/frail” if they fulfilled at least one frailty criteria. In order to determine associations among variables and to control for other variables potentially affecting frailty syndrome (age, comorbidity index and previous chemotherapy treatment), we performed a logistic regression analysis. The receiver operating characteristic curve was performed to assess the sensitivity and specificity of the hormonal concentration to discriminate prefrail/frail versus non-frail individuals. Significant positive associations were observed between the severity of frailty syndrome and estrone, FSH and LH concentrations and the aromatase activity index in the blood (*p* < 0.05). Further research into the role of hormonal biomarkers should be evaluated in follow-up studies in order to recommend their use as suitable biomarkers of frailty syndrome in breast cancer patients.

## 1. Introduction

The increased incidence and improved diagnostic and therapeutic interventions over the past decades have led to a rise in the prevalence of oncological diseases among the older patients [1]. According to the World Cancer Observatory (2019) [2], breast cancer mortality has fallen by 30% in the last 20 years, although it continues to rank among the five leading causes of oncological mortality in Spain and worldwide [3]. The physiological changes occurring during aging processes increase the risk of malignancies, as well frailty syndrome, which represents a state of increased vulnerability resulting from aging-associated decline in reserve and function across multiple physiologic systems, such that the ability to cope with acute stressors is compromised [4]. This syndrome involves physical deterioration and the loss of functions due to the loss of physiological reserves in the process of aging [5] or as a consequence of some chronic diseases [6]. The onset of frailty syndrome may limit therapeutic options and prognosis due to its numerous consequences, such as a lack of adherence to treatment and increased risk of disability, dependence, comorbidity and death [6]. The pathophysiological basis of frailty syndrome includes several systems such as the immune, muscular and endocrine systems, with the latter including estrogens and androgen hormones [7].

Geriatric assessment in oncology patients in clinical practice are useful and are able to detect frail patients. The International Society of Geriatric Oncology has established recommendations on the performance of geriatric assessments: they can lead to the detection of previously unidentified problems and risks for which specific interventions can be applied and to the prediction of adverse outcomes (e.g., toxicity of a therapy or functional or cognitive impairment) [8]. The primary goal of geriatric assessment is to provide a comprehensive health assessment to guide specific interventions and help select the appropriate cancer treatment. The geriatric assessment includes at least assessment of functional status, cognition and mood. Regarding evaluation of the functional assessment, measurements of a frailty phenotype based on physical criteria (low grip strength, low energy, slowed walking speed, low physical activity and/or unintentional weight loss) proposed by Fried and co-workers could be useful to identify frail individuals [9].

Menopause and menopause-related characteristics has been reported to be a predisposing factor for frailty syndrome [10]. The dramatic decline in hormone concentrations observed in menopause may account for the increased prevalence of both frailty and sarcopenia in women compared to men [11,12]. Many studies found that blood testosterone concentrations, and free testosterone concentrations in particular, are significantly lower in frail individuals of both genders [13,14,15], although mixed results were reported in women [16]. Different changes in dehydroepiandrosterone (DHEA) levels in the blood have been also associated with frailty, with higher levels in men [17], and lower DHEA levels have been reported in the serum of frail subjects [18,19]. The concentration of estrogens, estradiol and estrone has been also associated with frailty syndrome in older men, although with mixed results in different studies [16,20,21,22]. In contrast, higher estradiol levels in older women have been associated with frailty [16]. The decline in estrogen production that occurs during menopause is also recognized as being one of the causes of the deterioration of women’s health, affecting the loss of muscle mass and strength, which represents the pillar of sarcopenia, a geriatric syndrome that partially overlaps with frailty syndrome [10]. A recent study performed in older women with early breast cancer identified a high prevalence of low muscle mass in the whole sample (almost 42.7%), almost equally distributed in frail and non-frail patients. However, when more stringent criteria that incorporate muscle function (such as EWGSOP 2) were used, only a limited proportion of patients (26%) could still be defined as “sarcopenic”. Indeed, severely sarcopenic patients were almost always classified as frail on the results of comprehensive geriatric assessment [23]. Both sarcopenia and frailty are affected by hormonal changes and tumor and cancer treatment [23,24,25].

Estrogens such as estradiol (predominant estrogen in premenopausal women) and estrone (predominant estrogen after menopause) in particular, and to a lesser extent androgens, have a direct influence on the onset, progression and cure of most cases of breast cancer [10,26]. The development of specific biomarkers for the detection of frailty is one of the most current fields of research for diagnosis, prognosis and treatment [27]. The study of the hormonal alterations associated with frailty syndrome in breast cancer patients could help clinical practice to detect frailty syndrome in at-risk groups and design strategies to prevent or delay its onset. The main objectives of the study were:

- To describe the presence of frailty syndrome and its severity in postmenopausal women with hormone-dependent breast cancer before starting hormonal therapy.
- To determine whether frailty syndrome is associated with alterations in geriatric assessment.
- To ascertain whether frailty syndrome and geriatric assessment are associated with hormonal gonadal alterations in androgens and estrogens, gonadotropins levels, and the aromatase activity index in the blood.

## 2. Methods

### 2.1. Design and Study Population

An analytical, cross-sectional study was performed. The study included postmenopausal women diagnosed with hormone-dependent early breast cancer who had undergone surgery to remove the tumor, radiotherapy and or chemotherapy and who were prior to receiving anti-estrogen treatment to prevent recurrence. The recruitment and evaluation were performed by the medical oncology service of the university hospital Doctor Peset in Valencia (Spain), during the last quarter of 2019 and first quarter of 2020. The exclusion criteria were refusal to participate by the women after being informed of the study objectives or failing to understand the Spanish language correctly. A total of 47 women agreed to participate and signed the informed consent. The study was approved by the clinical research ethics committee of the Hospital Doctor Peset university hospital (protocol code 57/18, approved on 5 July 2018).

### 2.2. Assessment of Frailty Syndrome

The assessment of the frailty syndrome was performed according to Fried’s five criteria, such that the presence of three of these criteria would indicate a frailty state [9]. The criteria were assessed as follows: (1) weight loss, defined as unintentional loss of 4.5 kg or more in the past year. (2) Self-reported exhaustion, assessed by the question “how often in the last week did you feel that everything you did involved fatigue?”, included on the Center for Epidemiological Studies of Depression (CES-D) Scale [28]. It was considered positive when the answer was “often” or “most of the time”. (3) Decreased physical activity, assessed with the International Physical Activity Questionnaire (IPAQ), validated in Spanish. The time spent during a week was divided into quintiles, and the lowest quintile (20% less active) was given a positive score for this criterion [29]. (4) Motor slowness, calculated using the 4.6-m walking gait test and considered positive when the participant was in the lowest quintile, after adjustment for height according to the “Short Physical Performance Battery” [30]. The cutoff was set at 7 s for participants with a height ≥1.59 m and at 6 s for participants with a height < 1.59 m. (5) Low muscle strength. Overall muscle condition was calculated based on handgrip strength measured with a digital hand-held dynamometer. The positive criterion was given to the last quintile recorded in the sample (20% less strength) [31]. Frailty defined using the frailty phenotype based on Fried’ criteria classified individuals as pre-frail if they fulfilled one or two criteria or frail if they fulfilled three or more. Participants that did not fulfill any criteria were classified as robust [9].

### 2.3. Geriatric Assessment

The cognitive status was assessed with the validated version of the Mini Cognitive Examination (MEC) [32]. Depressive symptoms were measured with the Geriatric Depression Scale (GDS-15) [33,34]. Functional status was assessed using the Barthel Index [35]. Nutritional status was assessed using the Spanish validated version of the Mini Nutritional Assessment (MNA) [36]. Body Mass Index (BMI) was adjusted for age according to the WHO recommendations. For participants under 65 years of age, BMI was calculated based on the following groups: <18.5 kg/m^2^ (underweight), 18.5–24.9 kg/m^2^ (normoweight), 25–29.9 kg/m^2^ (overweight) and ≥30 (obese). For participants older than 64 years, BMI was calculated based on the following groups: <16 kg/m^2^ (severe malnutrition), 16.1–18.4 kg/m^2^ (moderate malnutrition), 18.5–22 kg/m^2^ (underweight), 22.1–24.9 kg/m^2^ (normoweight), 25–29.9 kg/m^2^ (overweight) and ≥30 kg/m^2^ (obesity) [37]. Circumferences of the arm (<27 cm implies low muscle mass), calf (<32.5 cm implies low muscle mass), hip (>99cm implies high fat mass) and abdomen (>85.9 cm implies high fat mass) were also measured [38,39]. The Athens Insomnia Scale was used to evaluate sleep quality [40,41]. The Charlson index was used to assess the comorbidity index [42].

### 2.4. Measurement of Hormones in Plasma

Blood samples were collected at between 9–10 a.m., under fasting conditions. After collection, blood samples were centrifuged at room temperature at 1500 rpm for 5 min. The supernatant was stored in 1 mL aliquots and frozen at −20 °C until analytical analysis, which took place within 3 days of blood collection. The concentrations of FSH, LH, estradiol, progesterone, testosterone and dehydroepiandrosterone sulfate were measured by the chemiluminescent microparticle immunoassay (CMIA). The dihydrotestosterone, androstenedione and estrone concentrations were measured using the ELISA Kit (Cloud-Clone Corp. Houston, TX, USA ref. CC-IEA443Ge, CC-IEA456Ge and Arbor assays ref. AA-K031-H5, respectively). Hormone levels were extrapolated from the standard solution curve.

### 2.5. Aromatase Activity Index in Plasma

The aromatase (the enzyme transforming androgens into estrogens) activity index in plasma was evaluated by calculating the ratio between the concentrations of estrone over androstendione in the blood, as a suitable index of aromatase activity in post-menopausal women [43,44]. The concentrations of aromatase in plasma was measured by the ELISA Kit (Cloud-Clone Corp. Houston, USA, ref. SEC319Hu) are expressed as ng/mL.

### 2.6. Statistical Analysis

The sample size was calculated based on the number of patients with breast cancer who started hormonal adjuvant treatment over the course of a year in the Oncology Department of the hospital Doctor Peset (Valencia) which was 77 patients in 2018. The sample size of the study population was calculated and estimated using two series model correlation tests with G*Power 3.1.9.2 software (G*Power©, Dusseldorf, Germany). A moderate correlation coefficient of r = 0.4; a two-tailed hypothesis; an error of α = 0.05, with a confidence interval of 95% and β error = 20%, and power analysis of 1 − β = 0.80 were also considered. Since correlation coefficient values below 0.3 are considered to be weak, 0.3–0.7 are moderate and >0.7 are strong [45,46], in order to calculate sample size, we assumed a moderate value of the coefficient correlation of 0.4. A sample size of at least 44 individuals was therefore considered appropriate for this study. For the descriptive statistics, we calculated the frequency distribution for the descriptive study of the qualitative variables. For the quantitative (discrete) variables, we obtained measures of central tendency (arithmetic mean), dispersion (standard deviation (SD) and the range of values. For the bivariate analysis, we categorized patients into two groups: robust (no frailty criteria) and prefrail/frail patients (patients fulfilling at least one frailty criteria); we used non-parametric tests (U-Mann Whitman–Whitney U-test). Spearman’s correlation was used for the analysis of two quantitative (discrete) variables. The statistical relationship between two categorical variables was studied by means of Pearson’s Chi-square test. A logistic regression analysis was used to try to make a predictive model in order to determine associations with the variables identified in bivariate analyses by controlling for other potential confounding variables. Logistic regression can be used to evaluate several factors simultaneously that are presumably related in some way (or not) to the dependent variable (presence or not of frailty criteria). The logistic regression analysis makes it possible to obtain measures of association (odds ratio) for each variable adjusted for the others and to detect possible interactions between them and the effect studied. The significantly different discrimination accuracy of the predictive model for the biomarkers between robust and prefrail/frail patients was calculated using C-statistics (the area under the receiver operating characteristic curve; AUC). The confidence level used for all the statistics was 95%, with a statistical significance (*p*) <0.05. The data were analyzed using the IBM SPSS Statistics 24^®^ program.

## 3. Results

### 3.1. Sociodemographic and Clinical Data

The sociodemographic data, clinical data related to breast cancer and comorbidity burden are summarized in Table 1. Forty-seven oncological patients were included, who were those who presented a TMN staging with a local stage I (2.1%), early regional stage II (72.1%) or late regional stage III (25.5%), with lymph node involvement and without the presence of distant metastasis, for which they received treatment with either conservative surgery (91.6%) or mastectomy (8.4%), as well as previous chemotherapy or radiotherapy (Table 1). Regarding the patients that received previous chemotherapy, the regimens were four patients who were treated with a chemotherapy schedule consisting of 4 cycles of chemotherapy based on doxorubicin (60 mg/m^2^)—cyclophosphamide (600 mg/m^2^) every 3 weeks and 12 weekly cycles of paclitaxel (80 mg/m^2^) [47,48]. Two patients were treated with liposomal doxorubicin (60 mg/m^2^)—cyclophosphamide (600 mg/m^2^) every 21 days [49]. The adjuvant chemotherapy regimen consisting of four cycles of docetaxel (75 mg/m^2^) and cyclophosphamide (600 mg/m^2^) administered every 3 weeks was performed in the remaining 2 patients [50]. Treatment with an anti-Her2 antibody was initiated in patients with HER2+ histology (four patients): two patients received trastuzumab (8 mg/kg initially and subsequently 6 mg/kg) and the other two received pertuzumab (840 mg initially and subsequently 420 mg) once every 3 weeks [51,52].

The mean age of the participants was 66.8 ± 1.3 (SEM) (age range 52–83 years), and all were community-dwelling women. As for marital status, they were married (48.9%), divorced (14.9%), separated (4.3%), single (8.5%) and widowed (24.4%). Regarding histology analyses, most of the patients had a ductal carcinoma (97.9%) and papillary carcinoma (2.1%). For estrogen hormone receptor staining, they presented a mean of 93.1 ± 1.6 (SEM) (range 40–100) and progestogen of 62.5 ± 5.2 (0–100), and only 2 patients presented as HER2 positive with a mean of 1.3 ± 0.09 (SEM) (range 1–3). The value of the proliferation marker Ki67 indicated a mean of 15.4 ± 2.03 (SEM) (range 1–60), i.e., they presented proliferative activity. As for the number of daily drugs, the table shows a mean of 3.2 ± 0.3 (range 0–11). The Charlson index adjusted for age indicated a mean index of 2.5 ± 0.1 (SEM) (range 2–5). The mean BMI of the participants was 28.9 ± 0.8 (SEM) (range 18.7–45). A total of 68.1% had received previous chemotherapy treatment, while 31.9% had not. However, most of the patients had received previous radiotherapy treatment (93.6%).

### 3.2. Evaluation of Frailty Syndrome

The mean number of fulfilled frailty criteria in the sample was 0.96 ± 0.12 (SEM) (range 0 to 3). The frequency of each of the five criteria is specified in Table 2. Fifteen women did not fulfil any criteria for frailty syndrome (31.9%), 21 (44.7%) fulfilled one frailty criterion, nine (19.1%) fulfilled two frailty criteria and two women (4.3%) fulfilled three frailty criteria. Based on Fried’s criteria (see Methods Section 2.3. Assessment of frailty syndrome), 31.9% of women were robust, 63.8% were pre-frail and 4.3% frail. Since the category of frail women was very small (N = 2) we pooled together prefrail and frail women in one group, and we called it the “prefrail/frail” group.

### 3.3. Evaluation of the Frailty Score and Socio-Demographic and Clinical Variables

There were no statistically significant differences in the age of women between the two groups (*p* = 0.86, Kruskal–Wallis test) nor any correlation between age and the frailty score (Rho = −0.109, *p* = 0.467, Spearman correlation test). There were no differences in the frailty score according to the civil status of the patients (*p* = 0.317, Kruskal–Wallis test). Frailty syndrome was not associated with the type of surgery (*p* = 0.128, Mann–Whitney test), nor with having received previous chemotherapy or not (*p* = 0.531). No significant correlations were found between the number of frailty criteria and staining for estrogens, progesterone or HER-2 and Ki67 in the histological analysis of breast tumors (*p* > 0.05 in all cases, Spearman correlation test). The values of Ki 67% were significantly and inversely correlated with the age of patients (Rho = −0.303, *p* = 0.043, Spearman correlation test).

There was no significant correlation between the frailty criteria and the Charlson index scale (Rho = 0.126, *p* = 0.398, Spearman correlation), the number of daily prescribed drugs (Rho = 0.112, *p* = 0.453, Spearman correlation), the Barthel index (Rho = −0.092, *p* = 0.544, Spearman correlation), depressive symptoms (Rho = 0.215, *p* = 0.147, Spearman correlation) or insomnia symptoms (Rho = 0.015, *p* = 0.942, Spearman correlation). In contrast, there was a significant inverse correlation between the frailty score and cognitive function (Rho = −0.314, *p* = 0.033, Spearman correlation). There were no significant correlations between frailty syndrome and nutritional assessment (Rho = −0113, *p* = 0.451, Spearman correlation) or anthropometric measurements.

### 3.4. Frailty Syndrome and the Concentration of Androgens and Estrogens and the Aromatase Activity Index in the Blood

There were significant (*p* < 0.05, Mann–Whitney test) differences between hormone concentrations in the blood between women fulfilling at least one frail criteria (“prefrail/frail” group) (N = 32) and those who were robust (N = 15) for FSH, LH, estrone concentration and the aromatase activity index in the blood. In contrast, no significant differences were observed for the concentration of progesterone, estradiol testosterone, dehydroepiandrosterone, androstenedione and dihydrotestosterone (Table 3).

**Figure 1:**
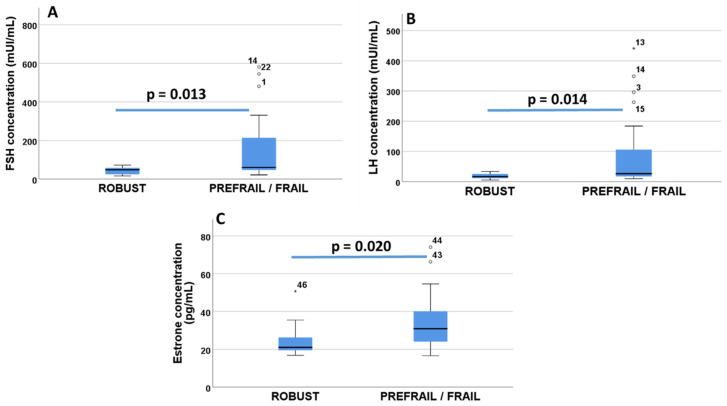
Differences between FSH (**A**), LH (**B**) and estrone (**C**) levels in non-frail (robust) and prefrail/frail women. “Robust” women (N = 15) did not fulfil any frailty criteria and ”prefrail/frail” (N = 32) was based on Fried’s classification of physical phenotype of frailty syndrome [9] (see also Methods Section 2.3. Assessment of frailty syndrome).

There was a significant direct correlation between frailty score and the blood concentration of FSH (Rho = 0.348, *p* = 0.017, Spearman correlation), LH (Rho = 0.012, *p* = 0.014, Spearman correlation) and estrone (Rho = 0.018, *p* = 0.020, Spearman correlation) but no significant correlations with the other hormones. By categorizing patients as robust (0 frailty criteria) or prefrail/frail (with one or more frailty criteria), the concentration of FSH, LH and estrone was significantly higher in the prefrail/frail group compared to the robust group (Figure 1A–C). There was no significant correlation between the number of frailty criteria and the concentration of the hormone estradiol, which was detected only in 12 out of 47 women (26.1% of the sample), and in these patients, it was not significantly correlated with the frailty syndrome score (Rho = 0.223; *p* = 0.485, Spearman correlation). The estradiol concentration may or may not be present in postmenopausal women.

**Figure 2:**
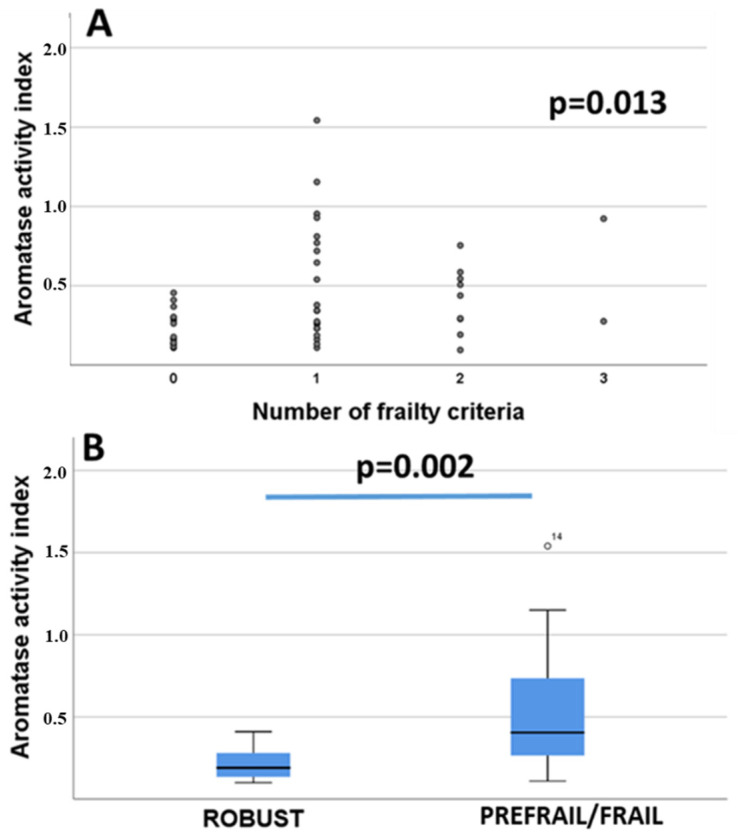
Aromatase activity index in non-frail (robust) and prefrail/frail women. (**A**): Correlation between number of fulfilled frailty criteria and aromatase activity index in blood; (**B**): Differences of aromatase activity index in robust and prefrail/frail patients. “Robust” women (N = 15) did not fulfil any frailty criteria and “prefrail/frail” (N = 32) were based on Fried’s classification of physical phenotype of frailty syndrome [9] (see also Methods Section 2.3. Assessment of frailty syndrome).

There was a significant direct correction between the frailty score and the aromatase activity index in the blood (Rho = 0.479, *p* = 0.013, Spearman correlation) (Figure 2A). When categorizing patients as robust (0 frailty criteria) and prefrail/frail (with one or more frailty criteria), the aromatase activity index was significantly higher in prefrail/frail compared to robust women (*p* = 0.002, Mann–Whitney test) (Figure 2B). There were no significant differences in the concentration of aromatase in plasma between prefrail/frail and non-frail women (*p* = 0.611, Mann–Whitney test; prefrail/frail women: 3.0 ± 0.5 ng/mL; non-frail: 3.4 ± 0.7 ng/mL).

A logistic regression analysis was used to determine associations with the variables identified in the bivariate analyses by controlling for other potential confounding variables (age, previous chemotherapy treatment, Charlson comorbidity index, hormones concentration and the aromatase activity index in the blood). By selecting as the dependent variable the dichotomous variable of “presence (prefrail/frail group) or not (robust group) of any frailty criteria”, we found significant effects for the aromatase activity index (*p* = 0.044), OR = 0.951 (95% CI 0.839–0.989), and for estrone concentration (*p* = 0.043), OR = 1.107 (95% CI 1.003–1.222). Other variables did not show a significant effect (Table 4).

**Figure 3:**
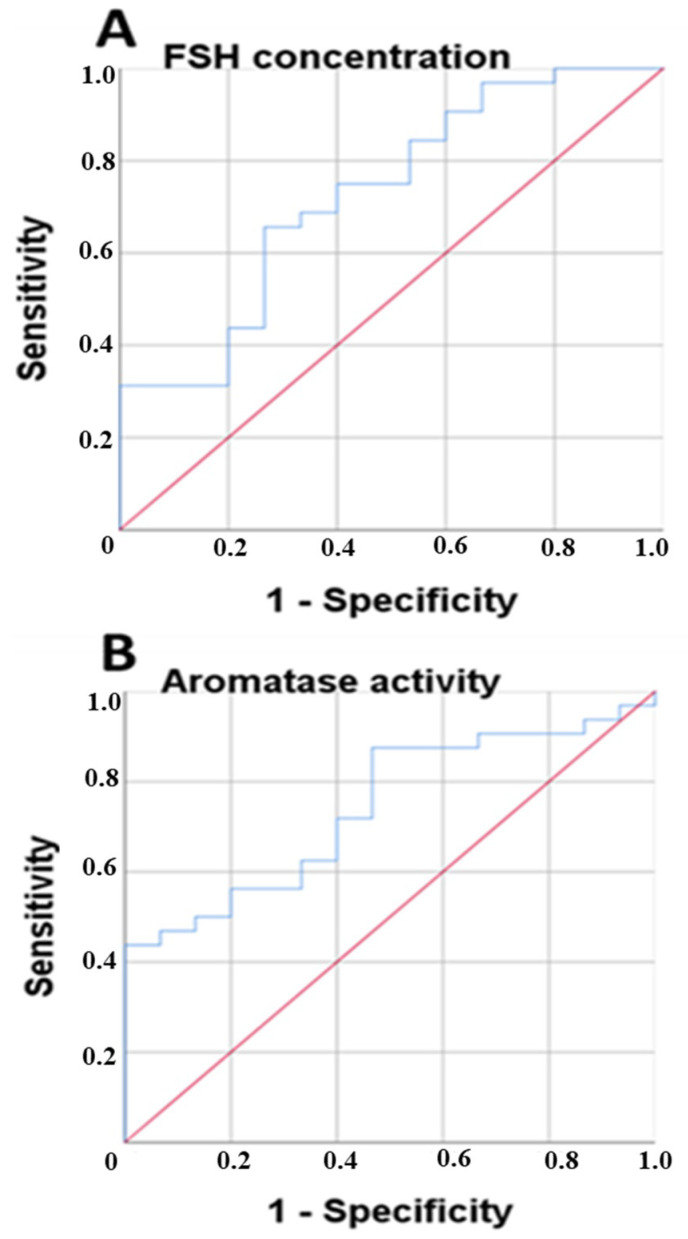
The receiver operating characteristic curve (ROC) for the FSH concentration in the blood (**A**) and the aromatase activity index in the blood (**B**) and the ability to discriminate between prefrail/frail and robust patients. “Robust” women (N = 15) did not fulfil any frailty criteria and ”frail” (N = 32) was based on Fried’s classification of physical phenotype of frailty syndrome [9] (see also Methods Section 2.3. Assessment of frailty syndrome).

Next, we performed a receiver operating characteristic curve (ROC) as a useful tool for evaluating the diagnostic power of these biomarkers to detect frailty syndrome categorized as robust and prefrail/frail (women fulfilling at least one criterion of frailty syndrome). This analysis provided an exhaustive look at the trend of sensitivity over all cutoffs and thus provided information about the relationship between the sensitivity and the specificity of FSH, LH and the aromatase activity index in the blood for the diagnosis of frailty (robust versus prefrail/frail patients) (Figure 3).

For the FSH concentration in the blood, the area under the curve value was 0.660, with a 95% CI of 0.499–0.822 (Figure 3A) with acceptable values, and the cut-off value of 55.2 had a sensitivity of 70.8% and a specificity of 39.1%. For the LH concentration, the area under the curve value was 0.581, with a 95% CI of 0.414–0.747, with the cut-off value of 22.8, which had a sensitivity of 54.2% and a specificity of 39.1%. For the estrone concentration, the area under the curve value was 0.582, with a 95% CI of 0.416–0.747, with a cut-off value of 23.2, and it had a sensitivity of 75% and a specificity of 56.5%. For the aromatase activity index, the area under the curve value was 0.761, with a 95% CI of 0.624–0.897, and the cut-off value of 20.83 had a sensitivity of 87% and a specificity of 54.2%.

## 4. Discussion

In our study, we identified approx. (31.9%) one third of women as robust and not fulfilling any frailty criteria. When considering the frail group definition based on Fried’s criteria (i.e., 3 or more criteria fulfilled), only 4.3% of women were frail, and the majority (63.8%) were prefrail (i.e., one or two frailty criteria fulfilled). We pooled together prefrail and frail women in one group called “prefrail/frail”, because the category of frail women was very small (N = 2). The rate of prevalence of frailty in our study was lower compared to the data obtained for the Fried frailty syndrome prevalence (9.7% of frail individuals) in the community-dwelling individuals enrolled in the Cardiovascular Health Study [9]. However, the mean age of participants enrolled in the present study (mean 66.8 years old) was lower compared to the population of the Cardiovascular Health Study, which could have contributed to the low prevalence of the frail group (those fulfilling three or more frailty criteria). The prevalence of frailty has been estimated at around 6.9% in community-dwelling older adults in the United States [53], and in a population-based study in Italy, its prevalence in women aged 65 years and over was 8.8% [54]. The Women’s Health and Aging Study I-II cohort study included 786 women aged 70–79 years old reported levels of 11.3% of frailty and 43.8% of prefrailty [55]. Compared to other studies in older women, the rate of frailty was similar or lower in our sample compared to older women without cancer, probably because the mean age of the older women in the present study was lower [56,57]. A recent meta-analysis [58] performed in patients with stage I to IV breast cancer reported a prevalence of frailty of around 40%, varying from 5% to 71% depending on the tumor stage (more frailty in advanced stages with respect to early stages of breast cancer) and the instrument used to detect frailty [59,60].

The pattern of prevalence of each of the frailty criteria in older community-dwelling women in Spain has been previously described, with the most prevalent being exhaustion (38.4%), followed by weakness (13.9%), low physical activity (11.8%), slow gait speed (9.5%) and involuntary weight loss (8.9%) [55]. In our study in women with early breast cancer, the rates and the distribution of individual frailty criterion were different as follows: low physical activity was 31.9%, involuntary weight loss was 23.4%, exhaustion was 21.3%, weakness was 10.7% and slow gait speed was 8.5%. The difference in the prevalence of the criterion of “involuntary weight loss” in breast cancer patients was significant compared to data from patients without cancer, in which this criterion of frailty was less prevalent [9,61]. Cancer is usually a traumatic experience for patients due to the various threats associated with the disease, including the diagnosis of a potentially fatal condition, complex treatment regimens, and the side effects resulting therefrom [62]. Cancer diagnosis has frequently been found to be related to psychiatric comorbidities such as depression and anxiety [63,64], and weight loss has been attributed possibly as a response either to the threat associated with being diagnosed with the disease or to patho-physiological mechanisms related to cancer. A meta-analysis of cancer in primary care reported a positive association between presenting to primary care with unexpected weight loss and a subsequent diagnosis of 10 different cancers [65]. The reason of the higher prevalence of the criterion “involuntary weight loss” in our sample compared to older women without cancer could be due to the reported effects, e.g., weight loss after cancer diagnosis, which takes place even when the disease is localized and could be due to a loss of appetite due to both physical and psychological reasons [66].

Overall, the variables included in the geriatric assessment did not correlate with frailty syndrome severity (i.e., the number of frailty criteria fulfilled) except for cognitive function, and, in fact, a significant inverse correlation was observed between frailty and cognitive function (the higher frailty syndrome, the lower cognitive functions).

These data replicate previous findings about the link between physical frailty and cognitive functions [67,68]. In the cross-sectional study by Jürschik et al. [69], the prevalence of frailty in community-dwelling people over 75 years of age was assessed using Fried’s criteria, as we used in the present study. The mean age of the participants was 81 years, and 60.3% were women. The prevalence of frailty among the participants was 9.6%. Cognitive impairment was detected in 20% of the frail subjects compared to 5.3% of the robust subjects. After logistic regression, cognitive impairment was significantly associated with frailty.

Several articles have explored the effect of frailty on specific cognitive domains, with two large-scale epidemiological studies standing out. The Rush Memory and Aging Study [70], conducted in 761 individuals with a mean age of 79 years, with a mean participant education of 14.5 years, a mean MMSE score of 28.4 and in which 76% of participants were female, showed that frailty was significantly associated with global cognition and perceptual speed [71]. In our study, the subdomain related with language in the MMSE was the subdomain significantly associated with frailty syndrome. The FIBRA study analyzed the frailty status according to the CHS frailty criteria in older individuals in Brazil [72] and found that frail participants had a significant impairment as measured by the MMSE, although the association with frailty syndrome was not only with the language subdomain, but also with the orientation and immediate memory subdomains of the MMSE.

We observed significant associations between frailty syndrome severity and the gonadotropins LH and FSH, estrone and the aromatase activity index in the blood. The associations between frailty syndrome and the gonadotropins FSH and LH were not related to the age of the patients, and they still remained after taking age into account as a potential confounder, suggesting it was not related to the time elapsed since menopause or the extent of hypogonadism. However, when controlling for other variables affecting frailty syndrome (previous chemotherapy treatment and Charlson comorbidity index), the significant associations between gonadotropins in the blood and the presence or not of frailty criteria were lost. A study performed in older men also reported a consistent association between frailty and gonadotropins in the blood [21]. In fully adjusted models, higher luteinizing hormone and follicle-stimulating hormones were positively related to worsening frailty syndrome in a 4-year follow-up study only in men but not in women [73]. We found no significant differences between testosterone levels, which replicate the findings of other studies reporting similar non-significant associations in women [16,18]. Very low levels of testosterone are observed in older women with ≥4 frailty criteria [16], and in our study, any women had this level of frailty, which may explain the lack of a significant association between frailty and testosterone. The association of free testosterone with frailty appeared confined to obese women [16]. Surgically, menopausal women had significantly lower total serum testosterone levels than naturally menopausal women, although they were not at a greater risk of frailty or death after adjusting for age, body mass index and the number of impaired instrumental activities of daily living [74].

Our study has been the first to report hormonal biomarkers as associated with prefrailty/frailty in post-menopausal women with breast cancer. The concentrations of FSH and LH show a moderate sensitivity and specificity to discriminate robust versus prefrail/frail women in our study, and it replicates the finding obtained in men [21,75]. The concentration of estrone, the major estrogen in post-menopausal women, was significantly increased in prefrail/frail women compared to robust ones. These data agree with other reports which showed higher estradiol associated with a higher degree of frailty syndrome [16] and higher estradiol in the blood, predicting a lower likelihood of improving frailty syndrome over time [73]. Consistent with our results, Schaap et al. [76] suggested a possible although not a significant association of higher estradiol in women included in the Longitudinal Aging Study Amsterdam. They also reported that women with high serum levels of estradiol had a higher rate of functional limitations. The lack of significance in their analyses could be due to a differential effect of estradiol on muscle strength and physical performance according to age, which they did not identify. Finally, another observational study reported no association between estrone, the primary estrogen present in postmenopausal women, and muscle mass and strength with poor physical performance and low muscle strength [77].

The increase in the estrogen concentration in the blood in frail postmenopausal women in the Toledo Study for Healthy Aging older was significantly associated with the high-sensitivity C-reactive protein concentration in the blood, suggesting the existence of physiopathological mechanisms connecting inflammation and estrogen concentration to frailty syndrome [16]. Confirming the results of the Toledo Study for Healthy Aging, our study also found that prefrail/frail women had increased estrone levels in the blood. Aromatase is a cytochrome p450 enzyme encoded by the CYP19A1 gene that converts androgens to estrogens and specifically converts testosterone and androstenedione to aromatic estrogens, estradiol and estrone, respectively [78]. Aromatase-facilitated estrogen production mainly occurs in the ovaries of premenopausal women, whereas in postmenopausal women, it takes place in peripheral tissues and especially in adipose tissue. Our study has been the first to report that an increase in the aromatase activity index (but not its concentration in plasma) is associated with frailty syndrome in post-menopausal women, with a reduction of the activity index in non-frail women compared to prefrail/frail ones, which goes in parallel with the changes observed in estrone (the product formed by its enzymatic activity). Among the biomarkers associated with frailty syndrome in breast cancer patients, the best profile is represented by the aromatase activity index, which showed a good sensitivity of 87% and a moderate specificity of 54.2% to discriminate between frail and non-frail patients. However, we must be cautious when interpreting the aromatase activity index as a biomarker of prefrailty/frailty in post-menopausal women until longitudinal studies are performed. In addition, we cannot exclude that the changes observed in hormone levels and the aromatase activity index may or may not be directly related to the development of frailty; there may be some intermediary factors, including inflammatory [79,80,81] and stress markers [82,83]. In fact, even in individuals with a low grade of frailty syndrome, such as prefrail individuals based on Fried’s criteria, frailty syndrome is associated with an increase in some peripheral inflammatory factors [84], and this aspect needs to be evaluated in post-menopausal women with breast cancer. In our study, most participants were prefrail based on the physical phenotype of Fried’s definition of frailty, e.g., they fulfilled one or two criteria of frailty, and future studies need to evaluate frailty syndrome in a population of women with breast cancer with higher odds of frailty. The fact that the presence of some of frailty criteria (prefrailty) is associated with a reduction in aromatase activity suggests that, as the first-line adjuvant treatment in post-menopausal women with breast cancer, aromatase inhibitor treatments may worsen frailty syndrome in these women. It should be pointed out that frailty syndrome based on Fried’s criteria, being composed of very general signs or symptoms, raises an “alert” about a possible problem related to increased vulnerability to stressors and a decrease in the physiological reserve. Appropriate therapeutic strategies, recommended in order to minimize the impact of frailty on the health of older woman with cancer, should start only after a comprehensive geriatric assessment that is capable of providing the required information supporting specific actions. The present study has some important limitations. First, the sample size was relatively small for a prevalent cancer, such as breast cancer. However, considering that the study was based on a single hospital center, the sample size was calculated based on the total number of patients with breast cancer (N = 77) who started hormonal adjuvant treatment over the course of a year; we think that a sample of 47 patients is quite acceptable for representing patients in our clinical context. Nevertheless, in our opinion, the study suggests future lines of research in this field of geriatric oncology. Second, although frailty has been conceptualized as a multidimensional geriatric syndrome, we evaluated it using the criteria proposed by Fried et al. [9], which are closely linked to physical condition. However, from our point of view these criteria have several advantages that make them suitable for the clinical setting, since they are objective, brief and easy to use [85]. The influence of previous chemotherapy regimens, however, did not show any significant difference in the study’s outcomes; we cannot rule out that enrolling a higher number of patients with previous chemotherapy could have led to some differences in the frailty syndrome or hormone measurements. Finally, the cross-sectional design of the study prevented us from evaluating the causality of the identified associations between prefrailty/frailty and inflammatory markers, and it did not allow us to rule out the possibility that these associations may be due to non-causal relationships.

## 5. Conclusions

The results of our study provide new evidence linking the presence of prefrailty/frailty in women with breast cancer to the alteration of hormonal changes in the blood and the aromatase activity index. In clinical practice, the identification of biomarkers, if confirmed in longitudinal studies, could be helpful in the early identification of post-menopausal women with breast cancer who are at risk of becoming prefrail or frail. These individuals would benefit from a comprehensive integral geriatric assessment, and treatment of the individual patient should be based on their health status regardimg frailty syndrome, according to the new directions in this field [86,87,88].

## Figures and Tables

**Table 1:** Sociodemographic and clinical characteristics.

Variables	Frequency % (Categorical Variables) or Mean and Standard Error of the Mean (Range Min-Max) (Discrete Variables)
Age (years)	66.8 ± 1.3 (52–83)
Marital status:	
Married	23 (48.9%)
Divorced	7 (14.9%)
Separated	2 (4.3%)
Single	4 (8.5%)
Widow	11 (24.4%)
Histology of tumor:	
Ductal carcinoma	46 (97.9%)
Lobular carcinoma	1 (2.1%)
Estrogen receptor staining (%)	93.1 ± 1.6 (40–100)
Progesterone receptor staining (%)	61.3 ± 0.09 (1–3)
HER2-positive staining (patients with 3 + staining in HER2: 4 patients)	2.5 ± 5.2 (0–10)
Ki67 mean values (%)	15.4 ± 2.03 (1–60)
Previous chemotherapy	
Yes	8 (17.0%)
No	39 (83.0%)
Previous radiotherapy	
Yes	44 (93.6%)
No	3 (6.4%)
Charlson comorbidity index	2.5 ± 0.1 (2–5)
Body mass index	28.9 ± 0.8 (18.7–45)

**Table 2:** Criteria of frailty syndrome and geriatric assessment.

	Prevalence (%)/Mean ± SEM
Frailty criterion: Involuntary weight loss	Yes 11 (23.4%)
	No 36 (76.6%)
Frailty criterion: Weakness	Yes 5 (10.7%)
	No 42 (89.3%)
Frailty criterion: Low physical activity	Yes 15 (31.9%)
	No 32 (68.1%)
Frailty criterion: Slow gait speed	Yes 4 (8.5%)
	No 43 (91.5%)
Frailty criterion: Low muscle strength	Yes 10 (21.3%)
	No 37 (78.7%)
Cognitive functions	28.3 ± 0.31 (range 22–30)
Activities of daily living (Barthel index)	97.8 ± 0.5 (range 90–100)
Insomnia symptoms (Athens scale)	2.9 ± 0.4 (range 0–9)
Depressive symptoms (Geriatric Depression Scale)	1.7 ± 0.3 (range 0–9)
Nutritional assessment (MNA scale)	26.9 ± 0.4 (range 20–30)

**Table 3:** Analysis of gonadotropins, androgens and estrogens concentrations and the aromatase activity index in the blood in prefrail/frail and non-frail patients. The significant (*p* < 0.05) differences between the two groups are shown in bold. “Robust” women (N = 15) did not fulfil any frailty criteria, and “prefrail/frail” (N = 32) was based on Fried’s classification of physical phenotype of frailty syndrome [9] (see also Methods Section 2.3. Assessment of frailty syndrome).

Hormones in Blood	Robust Patients (Mean ± Standard Deviation)	Prefrail/Frail Patients (Mean ± Standard Deviation)	*p* Values	Effect Size (Cohen’s d)
FSH (mUI/mL)	43.6 ± 5.05	186.2 ± 48.2	**0.013**	−0.628
LH (mUI/mL)	17.9 ± 2.2	79.03 ± 19.7	**0.014**	−0.658
Progesterone (ng/mL)	0.07 ± 0.01	0.07 ± 0.005	0.7	0.003
Estrone (pg/mL)	24.9 ± 2.28	34.3 ± 2.5	**0.02**	−0.72
Estradiol (pg/mL)	7.33 ± 0.9	7.68 ± 1.1	0.60	−0.062
Testosterone (ng/mL)	0.21 ± 0.02	0.30 ± 0.03	0.06	−0.548
Dehydroepiandrosterone (ug/dL)	103.2 ± 15.5	87.2 ± 9.54	0.50	0.285
Androstenedione (pg/mL)	123.8 ± 12.9	101.2 ± 11.6	0.15	0.367
Dihydrotestosterone (ng/mL)	0.151 ± 0.02	0.13 ± 0.01	0.54	0.271
Aromatase activity index	39.1 ± 4.6	31.4 ± 5.4	**0.03**	0.278

**Table 4:** Logistic regression model: variables associated with the outcome variable (presence or not of frailty criteria).

Variables	*p*-Value	OR	95% IC
Aromatase activity index	*p* = 0.043	0.951	0.839–0.989
Estrone	*p* = 0.034	1.107	1.003–1.222
Androstenedione	*p* = 0.169	0.990	0.975–1.004
Age	*p* = 0.450	0.964	0.877–1.060
FSH	*p* = 0.508	1.017	0.968–1.068
LH	*p* = 0.657	1.019	0.938–1.106
Previous chemotherapy treatment	*p* = 0.913	0.867	0.067–11.162
Charlson comorbidity index	*p* = 0.657	1.387	0.328–5.865

## Data Availability

The data presented in this study are available on request from the corresponding author for scientific purposes.
